# Formulation and Performance Analysis of Broadband and Narrowband OFDM-Based PLC Systems

**DOI:** 10.3390/s21010290

**Published:** 2021-01-04

**Authors:** Fausto García-Gangoso, Manuel Blanco-Velasco, Fernando Cruz-Roldán

**Affiliations:** Department of Teoría de la Señal y Comunicaciones, Escuela Politécnica Superior de la Universidad de Alcalá, 28805 Alcalá de Henares, Spain; fausto.garcia@edu.uah.es (F.G.-G.); manuel.blanco@uah.es (M.B.-V.)

**Keywords:** power line communications (PLC), orthogonal frequency-division multiplexing (OFDM), cyclic prefix (CP), windowing, overlap-and-add, bit error rate (BER), achievable data rate, Narrowband (NB), Broadband (BB), Internet-of-Things (IoT), smart home, smart grids

## Abstract

The aim of this paper is to formulate the physical layer of the broadband and narrowband power line communication (PLC) systems described in standards IEEE 1901 and IEEE 1901.2, which address new communication technologies over electrical networks for Smart Grid and Internet of Things applications. Specifically, this paper presents a mathematical formulation by means of matrices of a transmitter and receiver system based on windowed OFDM. The proposed formulation is essential for obtaining the input–output relation, as well as an analysis of the interference present in the system. It is very useful for simulating PLC systems using software designed to operate primarily on whole matrices and arrays, such as Matlab. In addition, it eases the analysis and design of different receiver configurations, simply by modifying or adding a matrix. Since the relevant standards only describe the blocks corresponding to the transmitter, and leave the set-up of the receiver open to the manufacturer, we analysed four different possible schemes that include window functions in different configurations. In simulations, the behaviour of each of these schemes is analysed in terms of bit error and achievable data rates using artificial and real noises.

## 1. Introduction

Power Line Communication (PLC) refers to the set of technologies that allow establishing electrical communications through power lines [1,2,3,4,5], and it could be a good choice for solving problems associated with the Internet of Things (IoT) [6,7]. IoT enables new functionality, such as consumers controlling and managing their power consumption. PLC provides an attractive technology for IoT connection, and it has become a competitive candidate technology to provide high-speed coverage [8,9,10]. Furthermore, PLC has proven its suitability in both broadband (BB) applications, such as interactive multimedia home incl. video-conferencing [11], and Narrowband (NB) communications, such as the Internet-of-things (IoT)/Smart Home [6,12] and Smart Grids [13]. Its great advantage is the ubiquity of such lines and, therefore, the cost savings that this implies for the deployment of a communications network. In contrast, power lines, not specifically designed as data transmission media, introduce a strong signal attenuation, as well as a large source of noises [14,15].

To make efficient use of the PLC channel by all classes of Power Line devices, the IEEE 1901 working group has released a set of standards focused on PLC, such as IEEE Standard for BB over Power Line Networks: Medium Access Control (MAC) and Physical Layer (PHY) Specifications (IEEE Std 1901-2010 [16]), and the IEEE Standard for Low-Frequency (less than 500 kHz) NB PLC for Smart Grid Applications (IEEE Std 1901.2-2013 [17]). More recently, BB PLC technologies for smart grid and for IoT have been specified, respectively, in IEEE 1901.1 [18] and in IEEE 1901a [19]. The above are part of the family of IEEE 1901 standards, and they address grid to utility meter, grid automation, electric vehicle to charging station, smart energy applications, lighting and solar panel PLC, or transportation platform (vehicle) applications. The standards specify the characteristics of the two first OSI layers (PHY and MAC) for, respectively, high- and low-speed communication devices. The BB system can reach data rates over 100 Mbps, using a bandwidth below 100 MHz, while the NB one offers data rates up to 500 kbps using a bandwidth below 500 kHz. These data rates can be increased for MIMO systems, where speeds of up to 2 Gbps can be achieved. In [5], coverage is increased and the throughput can be more than doubled upgrading from a SISO PLC to a 2×2 MIMO configuration. In [20], it is shown that if the full frequency range is used, HomePlug AV2 provides a 1 Gbps throughput in SISO configuration and 2 Gbps in a MIMO configuration.

To alleviate the adverse conditions introduced by a PLC channel, both standards deploy Orthogonal Frequency-Division Multiplexing (OFDM) as a modulation technique in the PHY. OFDM has demonstrated an intrinsic ability to overcome the adverse effects of transmission channels in modern communication systems, and an inherent adaptability to deal with noise [21,22]. However, OFDM is not free of shortcomings, such as high peak-to-average power ratio (PAPR), or a very large out-of-band (OOB) emission due to the sidelobes generated by the Fourier transform.

Both standards [16,17] recommend the use of cyclic prefix (CP) to maintain orthogonality between the OFDM subcarriers, thus avoiding or reducing intercarrier or intersymbol interference. Moreover, a phase shift between carriers is prescribed to diminish the PAPR. Additionally, a windowing process after performing the inverse discrete Fourier transform (IDFT) is also included, with the aim of reducing the OOB leakage of the signals to be transmitted [23,24,25,26]. The window function is left to the choice of the manufacturers, although a tapered window with side slopes defined by a step linear function is recommended. Finally, in order to meet the requirements of the specific regulations of each country regarding electromagnetic interference, the standards prescribe a tone mask that turns off certain subcarriers to comply with a predefined particular spectral mask.

The aim of this article is to formulate the digital front-end blocks of the PHY detailed in [16,17] and, in addition, to analyse and to compare the performance of four possible implementations (detailed in Table 1). Based on [27], we first provide a general matrix description of the windowed OFDM, but also including specific blocks defined in PLC systems that make the formulation applicable to BB and NB transceivers. As case studies, we provide the values of each matrix following the parameters defined in the PHY specifications of the BB and NB standards. Then, we analyse and compare the performance of each scheme, in terms of the bit error rate (BER) and achievable data rate.

The remainder of this paper is organized as follows. In Section 2, we present the system model and obtain the input–output relation. In Section 3, as a particular study-case, the formulation for the parameters deployed in the BB standard is presented. Moreover, some experimental results are presented. Section 4 does the same for NB systems, and finally, Section 5 provides some concluding remarks.

*Notation*: We use bold-face letters to indicate vectors (lower case) and matrices (upper case). $A^{T}$ represents the transpose of A. $I_{N}$ denotes the N×N identity matrix. The subscript is omitted whenever the size is clear from the context. 0 and 1 denote a matrix of zeros and ones, respectively.

## 2. System Model

**Figure 1:**
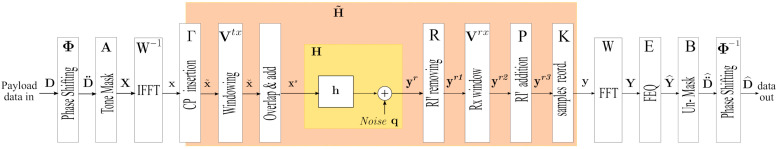
Simplified functional block diagram of a windowed OFDM PLC system.

Let us consider the windowed OFDM block diagram depicted in Figure 1 as a representative of some blocks of the PHY payload system proposed in both BB and NB PLC standards [16,17]. This is a simplified functional block diagram, in which the Tx side includes some stages deployed by both standards, while the Rx side is an authors’ proposal, since this is not specified in these standards. The functional blocks of this Figure are characterized by matrices, which are described in Table 2 and formulated below. The proposed matrix description is based on [27,28], and it includes some novelties, such as the phase shifting stage or the tone mask block. To the best of the authors’ knowledge, they have not been previously formulated using matrices, as well as their application to NB and BB PLC systems.

### 2.1. Transmitter

#### 2.1.1. Phase Shifting

Let us consider the vector $D_{M\times 1}=\left\{D_{0},D_{1},\ldots ,D_{M-1}\right\}$ that represents *M* digitally modulated symbols. After the modulation, the phase of each sub-carrier is shifted with respect to that of the other sub-carriers, with the aim of reducing the PAPR. The phase of the output signal, in radians, is obtained by multiplying the phase angle of the input signal by a factor of $\phi_{i}$. This block is represented in Figure 1 by Φ, this matrix being defined as

$$\Phi_{M}=diag\left(\phi \right),$$

where

$$\phi_{1\times M}=\left[e^{j\phi_{1}}\;e^{j\phi_{2}}\;\ldots \;e^{j\phi_{M}}\right],$$

where $\phi_{i}$ is a real number that defines a phase angle: its value is established in the standard for each active-carrier *i*. Thus, the resulting signal at the output of this block is given by

$${\ddot{D}}_{M\times 1}=\Phi_{M}\cdot D_{M\times 1},$$

where *M* is the number of active sub-carriers.

#### 2.1.2. Tone Mask

**Figure 2:**
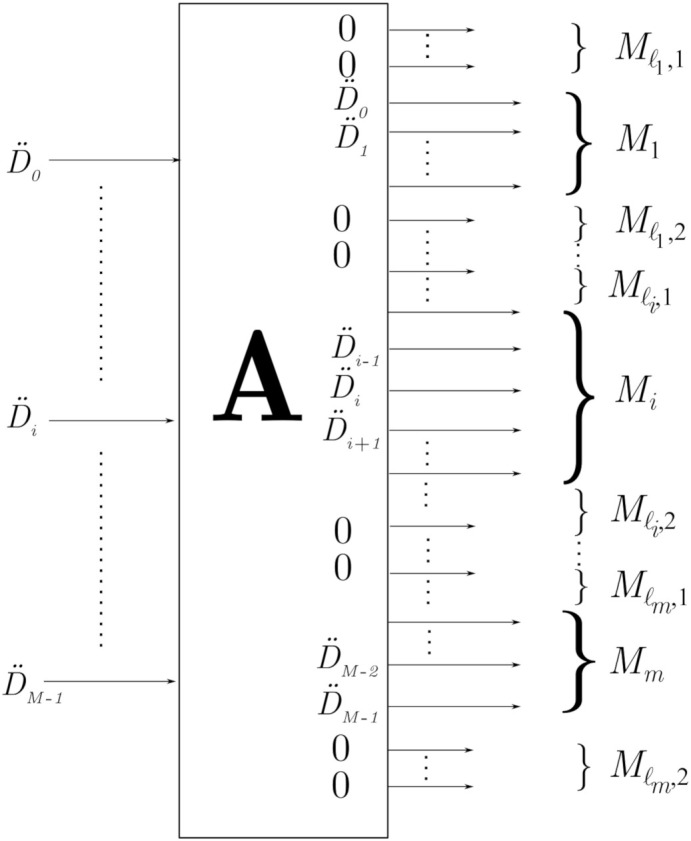
Active sub-carriers occupying non consecutive positions.

The standards also include a tone mask that disables certain (non-active) sub-carriers to reduce the effects of interference. This block is represented in Figure 1 by $A_{N\times M}$, and its goal is to accommodate the data ${\ddot{D}}_{M\times 1}={\left[{\ddot{D}}_{0},\ldots ,{\ddot{D}}_{M-1}\right]}^{T}$ of the *M* active sub-carriers within a total of N≥M sub-carriers deployed by the standard. The N−M inactive sub-carriers are usually set to zero. The result of this tone-masking operation can be expressed as follows:

$$X_{N\times 1}=\left[\begin{array}{c} X_{0}\\ X_{1}\\ \vdots \\ X_{N-1} \end{array}\right]=A_{N\times M}\cdot {\ddot{D}}_{M\times 1},$$

where A can be defined in two alternative ways:

(a) The active sub-carriers are allocated in consecutive positions. In this case, A is defined by
  $$A_{N\times M}=\left[\begin{array}{c} 0_{M_{1}\times M}\\ I_{M}\\ 0_{M_{2}\times M} \end{array}\right],$$
  where $M_{1}\geq 0$ is the number of inactive sub-carriers located at the beginning of the total set, and $M_{2}\geq 0$ is the number of those located at the end. One has $N=M_{1}+M+M_{2}$.
(b) The active sub-carriers are in non-consecutive positions, as depicted in Figure 2. In this case, A is defined by
  $$A_{N\times M}=\left[\begin{array}{cccc} A^{\ell_{1}} & 0 & \cdots & 0\\ 0 & A^{\ell_{2}} & \cdots & 0\\ \vdots & \vdots & \ddots & \vdots \\ 0 & 0 & \cdots & A^{\ell_{m}} \end{array}\right],$$
  where
  $$A_{\left(M_{\ell_{i},1}+M_{i}+M_{\ell_{i},2}\right)\times M_{i}}^{\ell_{i}}=\left[\begin{array}{c} 0_{M_{\ell_{i},1}\times M_{i}}\\ I_{M_{i}}\\ 0_{M_{\ell_{i},2}\times M_{i}} \end{array}\right],$$
  and
  $$\begin{array}{cc} \; & \ell_{act}=\sum_{i=1}^{m}M_{i},\\ \; & \ell_{inact}=\sum_{i=1}^{m}\left(M_{\ell_{i},1}+M_{\ell_{i},2}\right),\\ \; & N=\sum_{i=1}^{m}\left(M_{\ell_{i},1}+M_{i}+M_{\ell_{i},2}\right), \end{array}$$
  with $\ell_{act}$ and $\ell_{inact}$ being the number of active and inactive sub-carriers, respectively.

#### 2.1.3. The Inverse Discrete Fourier Transform (IDFT)

Next, an *N*-Point IDFT is applied to each input data vector X:

$$x_{N\times 1}={\left[x_{0},x_{1},\ldots ,x_{N-1}\right]}^{T}=W_{N}^{-1}\cdot X_{N\times 1},$$

where $W^{-1}$ represents the N×N IDFT matrix with elements ${\left[W^{-1}\right]}_{n,k}=\frac{1}{N}e^{j2\pi nk/N}$. This yields a stream of *N* samples in the time domain.

#### 2.1.4. Insertion of a Cyclic Prefix (CP)

**Figure 3:**
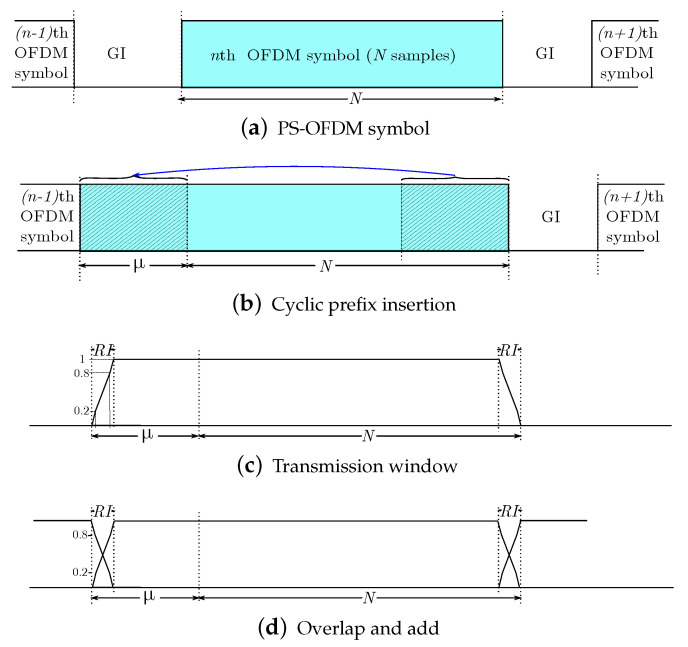
Prefixing, windowing and overlapping of an OFDM symbol in transmission.

The last μ samples of the symbol are appended to the beginning of each data vector. This operation, graphically represented in Figure 3b, is implemented by

$$\Gamma_{(N+\mu )\times N}=\left[\begin{array}{c} \begin{array}{cc} 0_{\mu \times (N-\mu )} & I_{\mu } \end{array}\\ I_{N} \end{array}\right],$$

in such a way that the output vector $\tilde{x}$ can be expressed as

$${\tilde{x}}_{(N+\mu )\times 1}=\Gamma_{(N+\mu )\times N}\cdot x_{N\times 1}.$$

#### 2.1.5. Tx Window

This block performs a multiplication, in the time domain, of each extended symbol with a window function, with the aim of reducing the OOB spectrum. To formulate this block, let us consider first the tapering window function defined by

$$v_{1\times (N+\mu )}^{tx}=\left[\begin{array}{ccc} v_{1\times RI}^{tr}\; & 1_{1\times ((N+\mu )-2RI)}\; & v_{1\times RI}^{tf} \end{array}\right],$$

where RI denotes the length of the roll-off interval, and $v^{tr}$ and $v^{tf}$ represent, respectively, the rising and falling slopes of the transmitter window, the elements of which satisfy

$$v_{1\times RI}^{tf}\left[n\right]=v_{1\times RI}^{tr}\left[-n+N+\mu -1\right],$$

for N+μ−RI≤n≤N+μ−1. With the above, we construct a new diagonal matrix given by

$$V_{\left(N+\mu \right)}^{tx}=diag\left\{v_{1\times (N+\mu )}^{tx}\right\}.$$

As a result, the output signal can be expressed as

$${\overset{˘}{x}}_{(N+\mu )\times 1}=V_{N+\mu }^{tx}\cdot {\tilde{x}}_{(N+\mu )\times 1}.$$

#### 2.1.6. Overlapping and Adding

This process consists of overlapping and adding RI samples of the *n*th symbol with the RI samples of the previous one, as depicted in Figure 3d. The main goal of this operation is to shorten the extra time domain overhead resulting from the CP insertion. This block will be formulated next, jointly with the channel convolution.

### 2.2. Channel

The conveyed signal $x^{s}$ is transmitted over the PLC channel, and becomes contaminated by PLC noise. The number of transmitted data vectors that affect the received signal is M+1 [27,28], where

(1) $$M\overset{\Delta }{\;=}\left\lceil \frac{\nu +RI}{N+\mu -RI}\right\rceil .$$

Then, the received signal is given by

$$y_{(N+\mu -RI)\times 1}^{r}\left[l\right]=\sum_{m=0}^{M}H_{\left(N+\mu -RI)\times (N+\mu \right)}^{\left(-m\right)}\cdot x_{(N+\mu )\times 1}^{s}\left[l-m\right]+q_{(N+\mu )\times 1}\left[l\right],$$

where $H^{\left(-m\right)}$ has entries [27,28]

(2) $${\left[H^{\left(-m\right)}\right]}_{b,c}\overset{\Delta }{\;=}\left\{\begin{array}{cc} 0, & mN_{0}+b-c<0,\\ h_{mN_{0}+b-c}, & 0\leq mN_{0}+b-c\leq \nu ,\\ 0, & mN_{0}+b-c>\nu , \end{array}\right.$$

with 0≤b≤N+μ−RI−1, 0≤c≤N+μ−1, $N_{0}=N+\mu -RI$, and q represents the channel noise.

### 2.3. Receiver

#### 2.3.1. RI Removal

**Figure 4:**
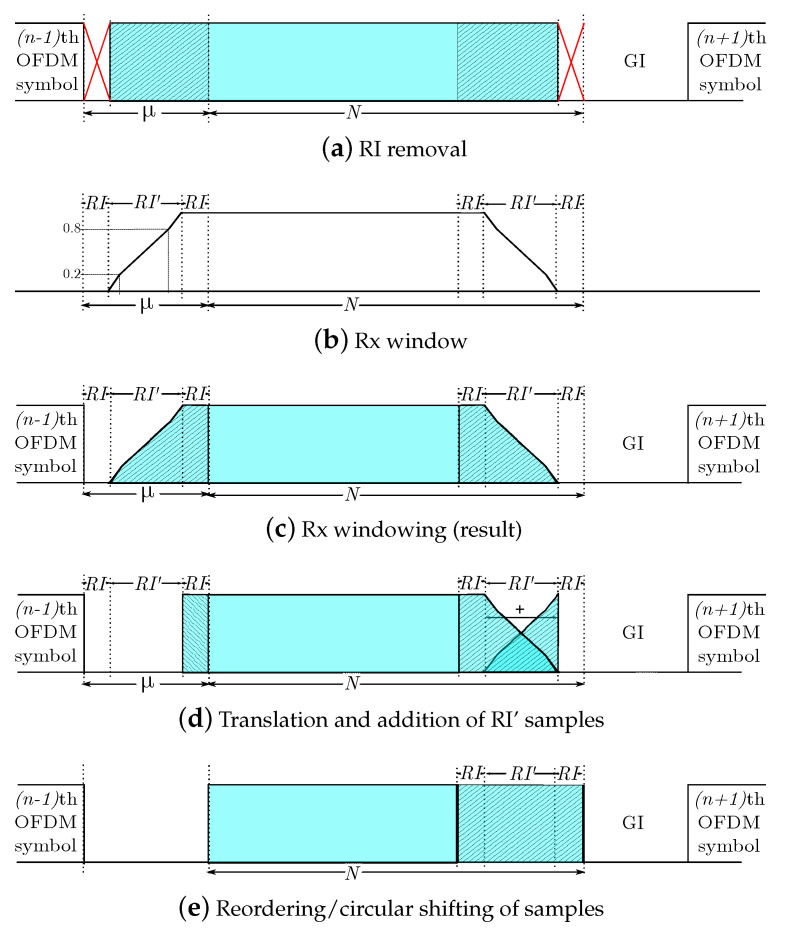
Operations for each OFDM symbol in reception.

The first RI samples, which correspond with the overlapped part of the symbol, are directly taken out (see Figure 4a), since these samples are not required to reconstruct the original symbol. This process can be formulated as

$$y_{(N+RI^{\prime })\times 1}^{r1}=R_{\left(N+RI^{\prime }\right)\times \left(N+\mu -RI\right)}\cdot y_{(N+\mu -RI)\times 1}^{r},$$

where

$$R_{\left(N+RI^{\prime }\right)\times \left(N+\mu -RI\right)}=\left[\begin{array}{cc} 0_{\left(N+RI^{\prime }\right)\times \left(\mu -RI-RI^{\prime }\right)} & I_{N+RI^{\prime }} \end{array}\right],$$

and $RI^{\prime }$ is the roll-off interval of the receiver window, defined next. Notice that RI=0 in the absence of a Tx window, and $RI^{\prime }=0$ in the absence of an Rx window.

#### 2.3.2. Rx Windowing

Many authors have pointed out the advantages of performing windowing in reception, e.g., to reduce the interference from other users, even though this operation was not carried out in transmission [21,22,29]. This block can be formulated similarly to its counterpart in the transmitter, i.e., the resulting signal is given by

$$y_{(N+RI^{\prime })\times 1}^{r2}=V_{\left(N+RI^{\prime }\right)}^{rx}\cdot y_{(N+RI^{\prime })\times 1}^{r1},$$

where

$$V_{\left(N+RI^{\prime }\right)}^{rx}=diag\left\{v_{1\times (N+RI^{\prime })}^{rx}\right\},$$

with

$$v_{1\times (N+RI^{\prime })}^{rx}=\left[\begin{array}{ccc} v_{1\times RI^{\prime }}^{rr} & 1_{1\times (N-RI^{\prime })} & v_{1\times RI^{\prime }}^{rf} \end{array}\right],$$

and $v^{rr}$ and $v^{rf}$ the rising and falling slopes of the receiver window, while $RI^{\prime }$ is the Rx roll-off interval. Figure 4b depicts an Rx window for the value of $RI^{\prime }=\mu -2RI$.

#### 2.3.3. RI’ Displacement and Addition

To reconstruct the transmitted signal, it is necessary to add the first $RI^{\prime }$ samples with the last $RI^{\prime }$ ones, as depicted in Figure 4d. The resulting signal is given by

$$y_{N\times 1}^{r3}=P_{N\times (N+RI^{\prime })}\cdot y_{(N+RI^{\prime })\times 1}^{r2},$$

where

$$P_{N\times (N+RI^{\prime })}=\left[\begin{array}{c} 0_{\left(N-RI^{\prime }\right)\times RI^{\prime }}\\ I_{RI^{\prime }} \end{array}\begin{array}{c} I_{N} \end{array}\right].$$

#### 2.3.4. Samples Reordering

If needed, the following block relocates the first RI samples of $y_{N\times 1}^{r3}$ at the end of this signal, as depicted in Figure 4e. This operation basically consists of a circular shift, defined by the matrix K as follows:

$$K_{N\times N}=\left[\begin{array}{cc} 0_{(N-RI)\times RI} & I_{\left(N-RI\right)}\\ I_{RI} & 0_{\mathrm{RI}\times (N-\mathrm{RI})} \end{array}\right].$$

Notice that if there is no window at the TX unit, this matrix is the identity matrix. Hence,

(3) $$y_{N\times 1}=K_{N\times N}\cdot y_{N\times 1}^{r3}.$$

#### 2.3.5. The Discrete Fourier Transform (DFT)

This block calculates

$$Y_{N\times 1}=W_{N\times N}\cdot y_{N\times 1},$$

where W represents the N×N DFT matrix with elements ${\left[W\right]}_{n,k}=e^{-j2\pi nk/N}$.

#### 2.3.6. Frequency Domain Equalizer (FEQ)

This block, represented by E, corrects for the transmission channel effects.

#### 2.3.7. Rx Data Arrangement

The resulting data must be rearranged in its original order

$${\hat{\ddot{D}}}_{M\times 1}=B_{M\times N}\cdot Y_{N\times 1},$$

where

$$B_{M\times N}=A_{N\times M}^{T}.$$

#### 2.3.8. Phase Shifting

Finally, the phase of the recovered data must be modified so that they return to their original values. This operation is carried out by $\Phi^{-1}$.

### 2.4. Recovered Data

Taking into account all the matrices previously defined, the final resulting data vector can be obtained as

(4) $$\hat{D}=\;R_{x}\cdot H^{\left(m\right)}\cdot T_{x}\cdot D+R_{x}\cdot H^{\left(m\right)}\cdot q,$$

where

$$\begin{array}{cc} R_{x} & =\Phi^{-1}\cdot A^{T}\cdot E\cdot W\cdot K\cdot P\cdot V^{\mathrm{rx}}\cdot R,\\ T_{x} & =V^{tx}\cdot \Gamma \cdot W^{-1}\cdot A\cdot \Phi , \end{array}$$

and q is a noise vector. This input–output relation is used in our computer simulations to obtain the BER. Furthermore, considering the analysis presented in [27], and including the matrices that distinguish the windowed PLC system from the conventional OFDM, it is possible to calculate the powers of the signal, interference and noise, and from the previous ones, the signal-to-interference-plus-noise ratio and the achievable data rate.

## 3. Case Study-I: BB PLC

In this section, we focus our attention on the FFT PHY of IEEE Std 1901–2010 [16]. First, we will specify each previously defined matrix adapted to the parameters prescribed by the standard for the Tx unit, given in Table 3.

Furthermore, we propose some receiver configurations compatible with the one in the standard. Next, we briefly describe the simulated channel and the noise signal models used in our experiments. Then, we evaluate and compare the performance of each configuration in the four BB PLC scenarios shown in Table 4.

The evaluation is carried out in terms of BER and achievable data rate. For all the simulations, the BPSK mapping is employed, and perfect time and frequency synchronization at the receiver side are assumed. Our PLC channels are time-invariant and constant during each transmission, and they are perfectly known by the receiver. To correct for its effect, a zero forcing FEQ is included at the receiver.

### 3.1. Matrix Description

Next we indicate the concrete values taken by the transmitter matrices. First, the phase shifting block can be partially described by

$$\Phi_{917\times 917}=\left[\begin{array}{cccc} e^{j7(\pi /4)} & 0 & \cdots & 0\\ 0 & e^{j7(\pi /4)} & \cdots & 0\\ \vdots & \vdots & \ddots & \vdots \\ 0 & 0 & \cdots & e^{j6(\pi /4)} \end{array}\right].$$

For a complete description of this matrix, we refer the reader to [16].

The tone mask matrix is formulated as

$$A_{4096\times 917}=\left[\begin{array}{cccc} A_{\left(154\times 54\right)}^{1} & 0_{\left(154\times 47\right)} & 0_{\left(154\times 700\right)} & 0_{\left(154\times 116\right)}\\ 0_{\left(67\times 54\right)} & A_{\left(67\times 47\right)}^{2} & 0_{\left(67\times 700\right)} & 0_{\left(67\times 116\right)}\\ 0_{\left(801\times 54\right)} & 0_{\left(801\times 47\right)} & \ddots & 0_{\left(801\times 116\right)}\\ 0_{\left(3074\times 54\right)} & 0_{\left(3074\times 47\right)} & 0_{\left(3074\times 700\right)} & A_{\left(3074\times 116\right)}^{9} \end{array}\right]$$

The CP insertion matrix is given by

$$\Gamma_{5348\times 4096}=\left[\begin{array}{c} \begin{array}{cc} 0_{1252\times 2844} & I_{1252} \end{array}\\ I_{4096} \end{array}\right].$$

Regarding the windowing block, the window function is defined in [16] as

(5) $$v\left[n\right]=\left\{\begin{array}{cc} v^{rise}\left[n\right], & 0\leq n\leq RI-1,\\ 1, & RI\leq n\leq N+GI-1,\\ v^{fall}\left[n\right], & N+GI\leq n\leq N+GI+RI-1, \end{array}\right.$$

where $v^{\mathrm{rise}}\left[n\right]$ and $v^{\mathrm{fall}}\left[n\right]$ include the components of the rise and fall segments, respectively, of the taper window, and GI is the guard interval (μ=GI+RI). Following (5), and assuming the window model suggested in [16], i.e., with linear piecewise sidelobes (three segments on each side), the rise segment can be defined as

$$\begin{array}{c} {\left[v_{1\times RI}^{tr}\right]}_{1,n}=\left\{\begin{array}{cc} \frac{0.2}{k_{1}}n, & 0\leq n\leq k_{1}-1,\\ 0.2+\frac{0.6}{k_{2}}\left(n-k_{1}\right), & k_{1}\leq n\leq k_{3}-1,\\ 0.8+\frac{0.2}{k_{1}}\left(n-k_{3}\right), & k_{3}\leq n\leq RI-1, \end{array}\right. \end{array}$$

where $v^{tr}$ represents the vector of *RI* samples of the rise sidelobe. Besides, $k_{1}=\left\lfloor 0.142RI\right\rfloor$, $k_{2}=\left\lceil 0.717RI\right\rceil$, and $k_{3}=\left\lfloor 0.859RI\right\rfloor$, in which $\left\lceil \cdot \right\rceil$ and $\left\lfloor \cdot \right\rfloor$ stand for the ceiling and floor functions, respectively, are the number of samples of each segment. Notice that $\frac{0.2}{k_{1}}$ is the slope of the first and third segments, while $\frac{0.6}{k_{2}}$ is the slope of the second segment. The scalars 0.2 and 0.6 represent the height (percentage over the unit) of each segment, see Figure 3c. These values, along with 0.8, have been obtained from the standard ([16], p. 1399). With the above, we have

$$V_{5348\times 5348}^{\mathrm{tx}}=\mathrm{diag}\;\left(\begin{array}{ccc} v_{1\times 496}^{tr}\; & 1_{1\times 4356}\; & v_{1\times 496}^{tf} \end{array}\right),$$

where

$$\begin{array}{c} {\left[v_{1\times 496}^{tr}\right]}_{1,n}=\left\{\begin{array}{cc} \frac{0.2}{70}n, & 0\leq n\leq 70-1,\\ \; & \\ 0.2+\frac{0.6}{356}\left(n-70\right), & 70\leq n\leq 426-1,\\ \; & \\ 0.8+\frac{0.2}{70}\left(n-426\right), & 426\leq n\leq 496-1, \end{array}\right. \end{array}$$

$${\left[v_{1\times 496}^{tf}\right]}_{1,n}=v_{1\times 496}^{tr}\left[-n+5347\right],\;4852\leq n\leq 5347,$$

being $v^{tf}$ the vector of *RI* samples of the fall sidelobe (see Figure 4b).

At the receiver, the matrices for each scheme of Table 4 are defined as follows:

(a) The transceiver only includes a window in the Tx unit. In this case, $RI^{\prime }=0$, $\mu -RI-RI^{\prime }=756$, and
  $$R_{4096\times 4852}=\left[\begin{array}{cc} 0_{4096\times 756} & I_{4096} \end{array}\right],$$
  $$V_{4096\times 4096}^{rx}=I_{4096},$$
  $$P_{4096}=I_{4096},$$
  $$K_{4096\times 4096}=\left[\begin{array}{cc} 0_{3600\times 496} & I_{3600}\\ I_{496} & 0_{496\times 3600} \end{array}\right].$$
(b) The transceiver only incorporates a window at the Rx unit. In this scheme, assuming $RI^{\prime }=496$, we have $\mu -RI-RI^{\prime }=756$, and
  $$R_{4592\times 5348}=\left[\begin{array}{cc} 0_{4592\times 756} & I_{4592} \end{array}\right],$$
  $$V_{4592}^{rx}=\mathrm{diag}\left(\begin{array}{ccc} v_{1\times 496}^{rr}\; & 1_{1\times 3600}\; & v_{1\times 496}^{rf} \end{array}\right),$$
  $$\begin{array}{c} {\left[v_{1\times 496}^{rr}\right]}_{1,n}=\left\{\begin{array}{cc} \frac{0.2}{71}n, & 0\leq n\leq 70,\\ \; & \\ 0.2+\frac{0.6}{355}\left(n-70\right), & 70\leq n\leq 222,\\ \; & \\ 0.8+\frac{0.2}{71}\left(n-431\right), & 431\leq n\leq 495, \end{array}\right. \end{array}$$
  $$v_{1\times 496}^{rf}\left[n\right]=v_{1\times 496}^{rr}\left[-n+4592\right],\;4096\leq n\leq 4591,$$
  $$P_{4096\times 4592}=\left[\begin{array}{c} 0_{3600\times 496}\\ I_{496} \end{array}\begin{array}{c} I_{4096} \end{array}\right],$$
  $$K_{4096\times 4096}=I_{4096}.$$
(c) The transceiver incorporates double window (Tx and Rx units). In this second scheme, we assume $RI^{\prime }=130$; thus $\mu -RI-RI^{\prime }=626$, and
  $$R_{4226\times 4852}=\left[\begin{array}{cc} 0_{4226\times 626} & I_{4226} \end{array}\right].$$
  If the following rising slope is assumed:
  $${\left[v_{1\times RI^{\prime }}^{rr}\right]}_{1,n}=\left\{\begin{array}{cc} \frac{0.2}{k_{1}}n, & 0\leq n\leq k_{1}-1,\\ \; & \\ 0.2+\frac{0.6}{k_{2}}\left(n-k_{1}\right), & k_{1}\leq n\leq k_{3}-1,\\ \; & \\ 0.8+\frac{0.2}{k_{1}}\left(n-k_{3}\right), & k_{3}\leq n\leq RI^{\prime }-1, \end{array}\right.$$
  $$v_{1\times RI^{\prime }}^{rf}\left[n\right]=v_{1\times RI^{\prime }}^{rr}\left[-n+N+RI^{\prime }\right],\;N\leq n\leq N+RI^{\prime }-1,$$
  where $k_{1}=\left\lceil 0.142RI^{\prime }\right\rceil$, $k_{2}=\left\lfloor 0.717RI^{\prime }\right\rfloor$, and $k_{3}=\left\lceil 0.857RI^{\prime }\right\rceil$, we have
  $$V_{4226}^{rx}=\mathrm{diag}\left(\begin{array}{ccc} v_{1\times 130}^{rr}\; & 1_{1\times 3966}\; & v_{1\times 130}^{rf} \end{array}\right),$$
  with
  $$\begin{array}{c} {\left[v_{1\times 130}^{rr}\right]}_{1,n}=\left\{\begin{array}{cc} \frac{0.2}{19}n, & 0\leq n\leq 18,\\ \; & \\ 0.2+\frac{0.6}{94}\left(n-19\right), & 19\leq n\leq 111,\\ \; & \\ 0.8+\frac{0.2}{19}\left(n-112\right), & 112\leq n\leq 129, \end{array}\right. \end{array}$$
  $$v_{1\times 130}^{rf}\left[n\right]=v_{1\times 130}^{rr}\left[-n+4226\right],\;4096\leq n\leq 4227.$$
  Finally,
  $$P_{4096\times 4226}=\left[\begin{array}{cc} \frac{0_{3966\times 130}}{I_{260}} & I_{4226} \end{array}\right],$$
  $$K_{4096\times 4096}=\left[\begin{array}{cc} 0_{3600\times 496} & I_{3600}\\ I_{496} & 0_{496\times 3600} \end{array}\right].$$
(d) The transceiver includes double window (Tx and Rx units) with $RI^{\prime }=260$, which is the maximum value possible for this scheme. Then, $\mu -RI-RI^{\prime }=496$, and
  $$R_{4356\times 4852}=\left[\begin{array}{cc} 0_{4356\times 496} & I_{4356} \end{array}\right],$$
  $$V_{4356}^{rx}=\mathrm{diag}\left(\begin{array}{ccc} v_{1\times 260}^{rr}\; & 1_{1\times 3836}\; & v_{1\times 260}^{rf} \end{array}\right),$$
  $$\begin{array}{c} {\left[v_{1\times 260}^{rr}\right]}_{1,n}=\left\{\begin{array}{cc} \frac{0.2}{37}n, & 0\leq n\leq 36,\\ \; & \\ 0.2+\frac{0.6}{186}\left(n-37\right), & 37\leq n\leq 222,\\ \; & \\ 0.8+\frac{0.2}{37}\left(n-223\right), & 223\leq n\leq 259, \end{array}\right. \end{array}$$
  $$v_{1\times 260}^{rf}\left[n\right]=v_{1\times 260}^{rr}\left[-n+4356\right],\;4096\leq n\leq 4355,$$
  $$P_{4096\times 4356}=\left[\begin{array}{cc} \frac{0_{3836\times 260}}{I_{260}} & I_{4096} \end{array}\right],$$
  $$K_{4096\times 4096}=\left[\begin{array}{cc} 0_{3836\times 260} & I_{3836}\\ I_{260} & 0_{260\times 3836} \end{array}\right].$$

Finally, the following matrix has the same values for the four configurations considered:

$$B_{917\times 4096}=A^{T}.$$

### 3.2. Channel and Noise Models

The channels herein considered are based on the statistical and multipath model proposed in [30,31] for in-home scenarios. Specifically, a bundle of 100 channels has been used in our experiments. Each of them is a different complex realization of Class 1 (high attenuation), Class 5 (medium attenuation) and Class 9 (low attenuation) channels [32]. These PLC channels are frequency selective, with 30 MHz of bandwidth and an order of 840 samples. The results next depicted are the averaged outcome of all of them.

According to ([16,33,34,35,36,37] Annex F.3.5.1), channels may have the following five types of noise: colored background noise, narrowband noise, impulsive periodic synchronous, impulsive periodic asynchronous and impulsive aperiodic. We employ the software described in [38] to generate sequences of noise of each type, as well as sequences composed by the sum of all of them. Concretely, two combinations of noise have been generated for each test: general backgroung noise (GBN), composed by the sum of colored background noise and narrowband noise, and ALL noises, i.e., the sum of the five noise types. The level of noise chosen for our simulations is“rand”, which means that the noise is generated with noise statistics randomly chosen between the “best” and “worst” levels. The authors claim that this yield the typical noise levels that can be expected to be found in an in-home PLC network scenario.

### 3.3. Simulations

**Figure 5:**
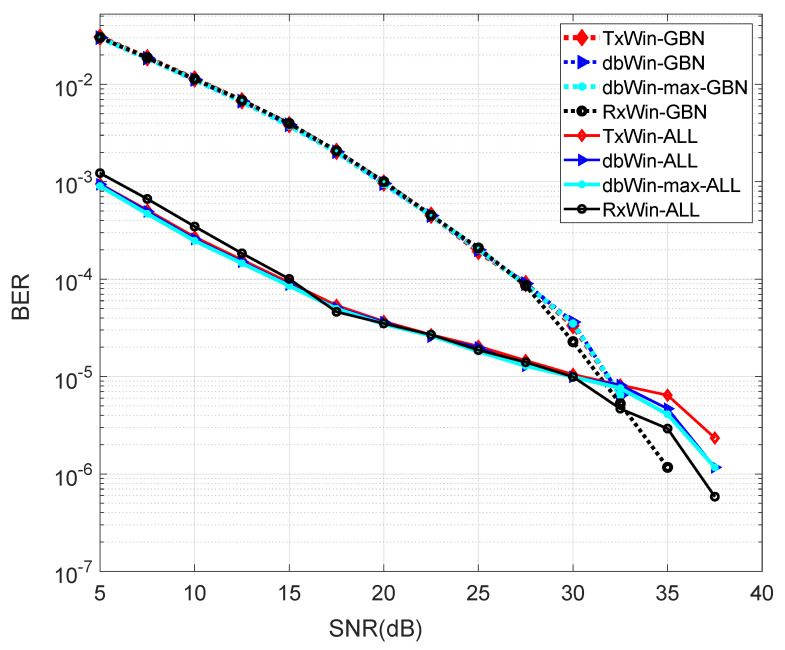
BER performance of four different IEEE 1901 BB systems.

The BER performance for the four schemes analysed has been evaluated using the above matrices, which are on the basis of a PHY 1901 OFDM transceiver like that depicted at Figure 1. The systems have been programmed in Matlab on the basis of the Monte Carlo method, with a loop of 1 × 10^3^ iterations. The BER obtained for the four different schemes are shown in Figure 5. As can be seen, there are practically no differences in the results for the different window schemes within each noise scenario (GBN, ALL). In addition, for almost the low SNR values, the sum of all PLC noises is a better scenario in terms of BER, than that in which GBN is present. There is a crossing point around 30 dB SNR that reflects a change in performance.

The achievable data rate has been obtained by applying the formulation in [27,28] but including the matrices of functional blocks, channels and noises previously defined. This data rate for the subcarrier *k* and using BPSK as primary modulation, is given by

$$R=f_{s}\sum_{k=0}^{N-1}\frac{N}{N+\mu }\cdot C\left(k\right),$$

being $f_{s}$ the sampling frequency and

$$C\left(k\right)=\frac{1}{2}\;\log_{2}\left(\frac{\mathrm{SINR}(k)}{\gamma }\right).$$

SINR stands for the signal and interference to noise ratio:

$$\mathrm{SINR}\left(k\right)=\frac{P_{\mathrm{signal}}\left(k\right)}{P_{\mathrm{ISI},\mathrm{ICI}}\left(k\right)+P_{\mathrm{noise}}\left(k\right)},$$

and γ is the modified SINR gap, which is defined, for a target symbol error rate (BER), as

$$\gamma ={\left(\frac{Q^{-1}\left(\mathrm{BER}/2\right)}{\sqrt{2}\pi }\right)}^{2}.$$

In [27,28], the expressions for the signal, interference and noise powers are derived. Furthermore, the impact of highly dispersive channels on OFDM, under finite-duration CIR with arbitrary length, is shown. We refer the reader to [27,28] and the references therein for a deeper analysis on the SINR and the achievable data rate calculation.

**Figure 6:**
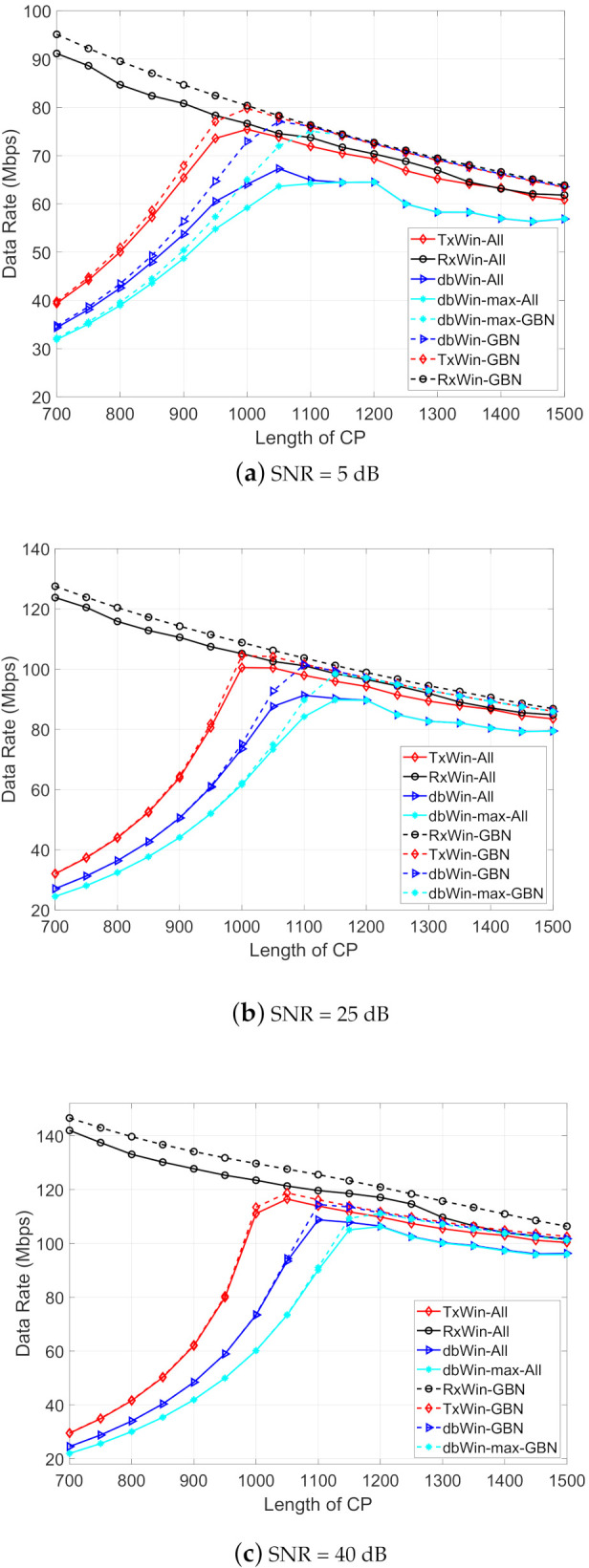
Achievable data rate as a function of the CP length for four IEEE 1901 BB systems.

Figure 6 shows the results for each scheme with the parameters from Table 3, and assuming three different SNR values: 5 dB, 25 dB and 40 dB. The γ value that corresponds to each SNR is obtained using the BER computed in the previous simulations. Observe that better results have been obtained, in all cases, for the scheme with only windowing at reception (RxWin). In contrast, the worst results are provided by the double windowing scheme. Regarding the type of PLC noise, a regular improvement in the data rate is observed for the case of ALL PLC noises while no appreciable difference is observed for the three SNR values in the case of GBN noise.

## 4. Case Study-II: NB PLC

In this section we pay attention to the NB FFT PHY described in IEEE Std 1901.2-2013 [17], specifically for the regulatory European band CENELEC-A. Again, four schemes, shown in Table 5, are analysed.

As in Section 3, the evaluation is carried out in terms of bit error rate (BER) and data rate. For all simulations, similar common parameters are assumed: BPSK mapping, perfect synchronization and channel knowledge at the receiver. Furthermore, a time-invariant channel response is assumed for the transmission process, and the FEQ is also designed following the zero-forcing criterion. Table 6 shows the main parameters specified in ([17] sub clause 6.3.2.1) for the CENELEC-A transmitter, which are employed in our simulations.

### 4.1. Matrix Description

At the transmitter, the first matrix characterizes the phase shifting block, and it can be partially described by

$$\Phi_{36\times 36}=\left[\begin{array}{cccc} e^{j2(\pi /8)} & 0 & \cdots & 0\\ 0 & e^{j1(\pi /8)} & \cdots & 0\\ \vdots & \vdots & \ddots & \vdots \\ 0 & 0 & \cdots & e^{j7(\pi /8)} \end{array}\right].$$

For a complete description of the phase factor matrix, we refer the reader to ([17], Tables 6–18).

The tone mask matrix is formulated as

$$A_{256\times 36}=\left[\begin{array}{c} 0_{22\times 36}\\ I_{36}\\ 0_{198\times 36} \end{array}\right],$$

The CP insertion matrix is given by

$$\Gamma_{286\times 256}=\left[\begin{array}{c} \begin{array}{cc} 0_{30\times 226} & I_{30} \end{array}\\ I_{256} \end{array}\right],$$

Since in [17] the design of the window function is left to the manufacturer, we here propose a piecewise linear window function, similar to that defined in Section 3. Hence, we have

(6) $$v\left[n\right]=\left\{\begin{array}{cc} v^{rise}\left[n\right], & 0\leq n\leq RI-1,\\ 1, & RI\leq n\leq N+GI-1,\\ v^{fall}\left[n\right], & N+GI\leq n\leq N+GI+RI-1, \end{array}\right.$$

where $v^{\mathrm{rise}}\left[n\right]$ and $v^{\mathrm{fall}}\left[n\right]$ include the components of the rise and fall segments, respectively, of a taper window, and GI is the guard interval (μ=GI+RI). The following rise segment satisfies the above definition:

$$\begin{array}{c} {\left[v_{1\times RI}^{tr}\right]}_{1,n}=\left\{\begin{array}{cc} \frac{0.2}{k_{1}}n, & 0\leq n\leq k_{1}-1,\\ \; & \\ 0.2+\frac{0.6}{k_{2}}\left(n-k_{1}\right), & k_{1}\leq n\leq k_{3}-1,\\ \; & \\ 0.8+\frac{0.2}{k_{1}}\left(n-k_{3}\right), & k_{3}\leq n\leq RI-1, \end{array}\right. \end{array}$$

where $k_{1}=\left\lfloor 0.142RI\right\rfloor$, $k_{2}=\left\lceil 0.717RI\right\rceil$, and $k_{3}=\left\lfloor 0.859RI\right\rfloor$, in which $\left\lceil \cdot \right\rceil$ and $\left\lfloor \cdot \right\rfloor$ stand for the ceiling and floor functions, respectively. With the above, we have

$$V_{286\times 286}^{tx}=\mathrm{diag}\left\{\begin{array}{ccc} v_{1\times 8}^{tr}\; & 1_{1\times 270}\; & v_{1\times 8}^{tf} \end{array}\right\},$$

where

$$\begin{array}{c} {\left[v_{1\times 8}^{tr}\right]}_{1,n}=\left\{\begin{array}{cc} \frac{0.2}{1}n, & n=0,\\ \; & \\ 0.2+\frac{0.6}{6}\left(n-1\right), & 1\leq n\leq 6,\\ \; & \\ 0.8+\frac{0.2}{1}\left(n-7\right), & n=7, \end{array}\right. \end{array}$$

$${\left[v_{1\times 8}^{tf}\right]}_{1,n}=v_{1\times 8}^{tr}\left[-n+285\right],\;278\leq n\leq 285,$$

At the receiver, the matrices are defined as follows:

(a) The transceiver only includes a window in the Tx unit. In this case, $RI^{\prime }=0$, $\mu -RI-RI^{\prime }=22$, and
  $$R_{256\times 278}=\left[\begin{array}{cc} 0_{256\times 22} & I_{256} \end{array}\right],$$
  $$V_{256\times 256}^{rx}=I_{256},$$
  $$P_{256}=I_{256},$$
  $$K_{256\times 256}=\left[\begin{array}{cc} 0_{248\times 8} & I_{248}\\ I_{8} & 0_{8\times 248} \end{array}\right].$$
(b) The transceiver only incorporates a window at the Rx unit. In this scheme, assuming $RI^{\prime }=8$, we have $\mu -RI-RI^{\prime }=22$, and
  $$R_{264\times 286}=\left[\begin{array}{cc} 0_{264\times 22} & I_{264} \end{array}\right],$$
  If the following rising slope is assumed:
  $$\begin{array}{c} {\left[v_{1\times RI^{\prime }}^{rr}\right]}_{1,n}=\left\{\begin{array}{cc} \frac{0.2}{k_{1}}n, & 0\leq n\leq k_{1}-1,\\ \; & \\ 0.2+\frac{0.6}{k_{2}}\left(n-k_{1}\right), & k_{1}\leq n\leq k_{3}-1,\\ \; & \\ 0.8+\frac{0.2}{k_{1}}\left(n-k_{3}\right), & k_{3}\leq n\leq RI^{\prime }-1, \end{array}\right. \end{array}$$
  $$v_{1\times RI^{\prime }}^{rf}\left[n\right]=v_{1\times RI^{\prime }}^{rr}\left[-n+N+RI^{\prime }\right],\;N\leq n\leq N+RI^{\prime }-1,$$
  where $k_{1}=\left\lceil 0.142RI^{\prime }\right\rceil$, $k_{2}=\left\lfloor 0.717RI^{\prime }\right\rfloor$, and $k_{3}=\left\lceil 0.857RI^{\prime }\right\rceil$, we have
  $$V_{264}^{rx}=\mathrm{diag}\left(\begin{array}{ccc} v_{1\times 8}^{rr}\; & 1_{1\times 248}\; & v_{1\times 8}^{rf} \end{array}\right),$$
  with
  $$\begin{array}{c} {\left[v_{1\times 8}^{rr}\right]}_{1,n}=\left\{\begin{array}{cc} \frac{0.2}{2}n, & 0\leq n\leq 1,\\ \; & \\ 0.2+\frac{0.6}{5}\left(n-2\right), & 2\leq n\leq 5,\\ \; & \\ 0.8+\frac{0.2}{2}\left(n-7\right), & 6\leq n\leq 7, \end{array}\right. \end{array}$$
  $$v_{1\times 8}^{rf}\left[n\right]=v_{1\times 8}^{rr}\left[-n+264\right],\;256\leq n\leq 263,$$
  Finally,
  $$P_{256\times 264}=\left[\begin{array}{c} 0_{248\times 8}\\ I_{8} \end{array}\begin{array}{c} I_{256} \end{array}\right],$$
  $$K_{256\times 256}=I_{256}.$$
(c) The transceiver incorporates double window (Tx and Rx units). In this second scheme, we assume $RI^{\prime }=7$; thus $\mu -RI-RI^{\prime }=15$, and
  $$R_{263\times 278}=\left[\begin{array}{cc} 0_{263\times 15} & I_{263} \end{array}\right],$$
  If the following rising slope is assumed:
  $${\left[v_{1\times RI^{\prime }}^{rr}\right]}_{1,n}=\left\{\begin{array}{cc} \frac{0.2}{k_{1}}n, & 0\leq n\leq k_{1}-1,\\ \; & \\ 0.2+\frac{0.6}{k_{2}}\left(n-k_{1}\right), & k_{1}\leq n\leq k_{3}-1,\\ \; & \\ 0.8+\frac{0.2}{k_{1}}\left(n-k_{3}\right), & k_{3}\leq n\leq RI^{\prime }-1, \end{array}\right.$$
  $$v_{1\times RI^{\prime }}^{rf}\left[n\right]=v_{1\times RI^{\prime }}^{rr}\left[-n+N+RI^{\prime }\right],\;N\leq n\leq N+RI^{\prime }-1,$$
  where $k_{1}=\left\lceil 0.142RI^{\prime }\right\rceil$, $k_{2}=\left\lfloor 0.717RI^{\prime }\right\rfloor$, and $k_{3}=\left\lceil 0.857RI^{\prime }\right\rceil$, we have
  $$V_{263}^{rx}=\mathrm{diag}\left(\begin{array}{ccc} v_{1\times 7}^{rr}\; & 1_{1\times 248}\; & v_{1\times 7}^{rf} \end{array}\right),$$
  with
  $$\begin{array}{c} {\left[v_{1\times 7}^{rr}\right]}_{1,n}=\left\{\begin{array}{cc} \frac{0.2}{2}n, & 0\leq n\leq 1,\\ \; & \\ 0.2+\frac{0.6}{10}\left(n-2\right), & 2\leq n\leq 11,\\ \; & \\ 0.8+\frac{0.2}{2}\left(n-12\right), & 12\leq n\leq 13, \end{array}\right. \end{array}$$
  $$v_{1\times 7}^{rf}\left[n\right]=v_{1\times 7}^{rr}\left[-n+270\right],\;256\leq n\leq 269,$$
  Finally,
  $$P_{256\times 263}=\left[\begin{array}{cc} \frac{0_{249\times 7}}{I_{7}} & I_{256} \end{array}\right],$$
  $$K_{256\times 256}=\left[\begin{array}{cc} 0_{248\times 8} & I_{248}\\ I_{8} & 0_{8\times 248} \end{array}\right].$$
(d) The transceiver incorporates double window (Tx and Rx units) but assuming $RI^{\prime }=14$, which is its maximum value possible for this scheme; thus $\mu -RI-RI^{\prime }=8$, and
  $$R_{270\times 278}=\left[\begin{array}{cc} 0_{270\times 8} & I_{270} \end{array}\right],$$
  $$V_{270}^{rx}=\mathrm{diag}\left(\begin{array}{ccc} v_{1\times 14}^{rr}\; & 1_{1\times 242}\; & v_{1\times 14}^{rf} \end{array}\right),$$
  $$\begin{array}{c} {\left[v_{1\times 14}^{rr}\right]}_{1,n}=\left\{\begin{array}{cc} \frac{0.2}{2}n, & 0\leq n\leq 1,\\ \; & \\ 0.2+\frac{0.6}{10}\left(n-2\right), & 2\leq n\leq 11,\\ \; & \\ 0.8+\frac{0.2}{2}\left(n-12\right), & 12\leq n\leq 13, \end{array}\right. \end{array}$$
  $$v_{1\times 14}^{rf}\left[n\right]=v_{1\times 14}^{rr}\left[-n+270\right],\;256\leq n\leq 269,$$
  $$P_{256\times 270}=\left[\begin{array}{cc} \frac{0_{242\times 14}}{I_{14}} & I_{256} \end{array}\right],$$
  $$K_{256\times 256}=\left[\begin{array}{cc} 0_{248\times 8} & I_{248}\\ I_{8} & 0_{8\times 248} \end{array}\right].$$

Finally, the following matrix is the same for all the receivers:

$$B_{36\times 256}=A^{T}.$$

### 4.2. Channel and Noise Models

In the NB case, a bundle of 100 NB PLC channels for in-home scenarios has been used following the specifications given in ([17] Annex D.2), based on the models proposed by [39,40]. The simulations here presented are the averaged outcome of all of them. As PLC NB noise, we have assumed four real noises collected from several usage scenarios (large industrial power plant and water chilling station, the living room of an apartment, laboratory space and student office area) and referred to as “Industrial”, “LivingRoom”, “LabSpace”, and “StudyOffice”, described in [41].

### 4.3. Simulations

The BER performance for the NB scenario was carried out in a similar way as the case BB, i.e., on the basis of the Monte Carlo method, with a loop of 1e3 iterations applied over the four schemes included in Table 5.

**Figure 7:**
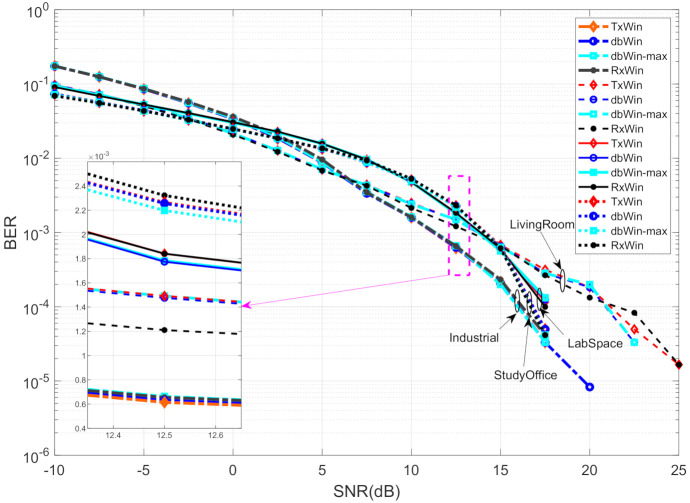
BER performance of four different IEEE 1901.2 NB systems.

The results are shown in Figure 7, and as in the BB case, no significant differences can be observed in terms of BER between the different PLC noises or between the four different schemes herein considered.

**Figure 8:**
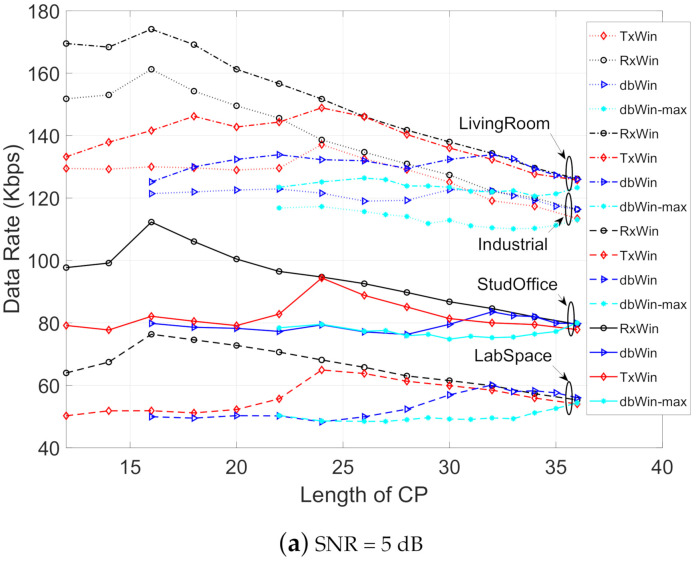

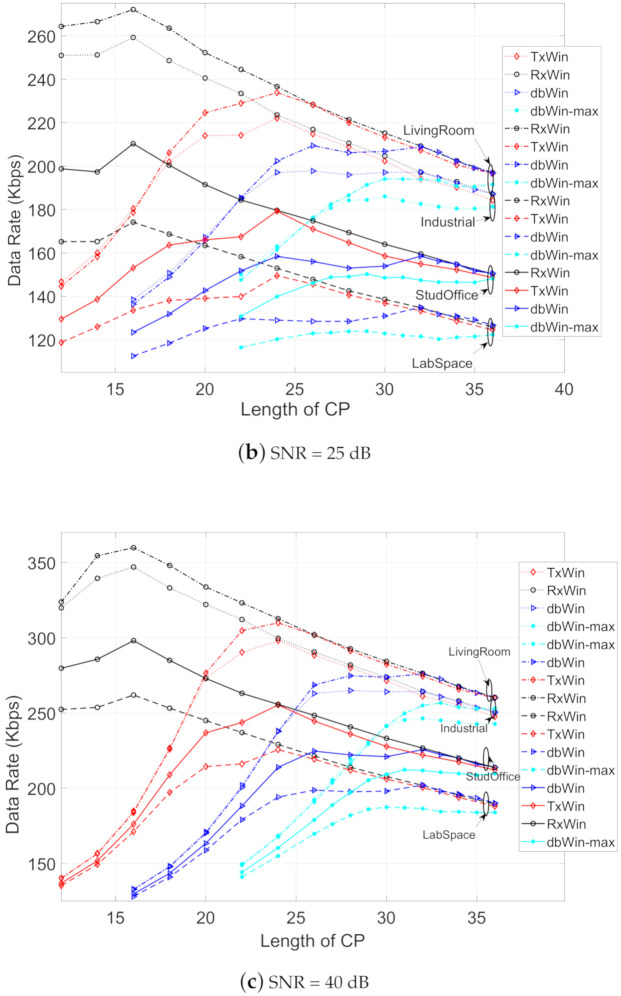
Achievable data rate as a function of the CP length for four IEEE 1901.2 NB systems.

Figure 8 depicts the achievable data rate obtained for the four IEEE 1901.2 schemes, considering the parameters of Table 6, different kinds of noise, and values of the SNR (5 dB, 25 dB and 40 dB). In all scenarios, the ranking of best achievable data rate performance is, in this order, the following: LivingRoom, Industrial, StudyOffice and LabSpace. Moreover, for each type of PLC noise, the best result is, for low lengths of CP (below 20 samples), for the scheme of windowing at reception (RxWin). The worst results are for the schemes of double windowing (dbWin and dbWin-max). For higher CP length values, a convergence of the obtained results is observed for each scheme.

## 5. Conclusions

In this paper, the digital front-end blocks of the PHY detailed in the BB and NB PLC standards are formulated. First, a generic and unified matrix description is derived. Then as case-studies, we focus our attention on IEEE 1901 (BB) and IEEE 1901.2 (NB), which are new communication technologies for smart home, smart building and IoT applications. The values of each of the matrices are provided according to the parameters described in these standards for the transmitter, and specific matrices are given that implement four compatible receivers that follow the defined recommendations. Specifically, four possible windowing schemes are proposed valid for both scenarios. Simulations of the channels and PLC noises were carried out. The results show that there are no appreciable differences in terms of BER between the different scenarios. However, as to the achievable data rate, we observe that the scheme of windowing only in reception outperforms the rest. In our simulations, the worst results have been obtained from the schemes with double windowing.

## Figures and Tables

**Table 1:** PLC schemes to be analysed.

#	Abbrev.	Comment
1	TxWin	1901 PLC system with windowing at transmission side.
2	RxWin	1901 PLC system with windowing at reception side.
3	dbWin	1901 PLC system with double windowing: at transmission and reception side.
4	dbWin-max	1901 PLC system double windowing: at transmission and reception side but with maximun admissible value of roll-off for the reception window.

**Table 2:** Matrices of each functional block.

Matrix	Description
Φ	Phase shifting
A	Tone mask
$W^{-1}$	Inverse discrete Fourier transform
Γ	CP insertion
$V^{tx}$	Transmitter (Tx) window
h	Channel impulse response
H	Channel matrix
R	Discards the samples of the Tx rolloff interval
$V^{rx}$	Receiver (Rx) window
P	Displaces and adds the samples of the Rx rolloff interval
K	Samples reordering
W	Discrete Fourier transform
E	Frequency domain equalizer
B	Inverse of the masking function A
$\Phi^{-1}$	Inverse phase shifting

**Table 3:** Tx parameters defined in the BB FFT PHY [16].

Parameter	Description	Value (Samples)
*N*	FFT size	4096
*M*	Active sub-carriers ^1^	917
μ	CP length (samples)	1252
RI	Samples of the Roll-off interval	496

^1^ Distributed in nine blocks according to the suggested tone mask given in ([16] sub clause 13.9.7).

**Table 4:** BB schemes to be analysed.

#	Abbrev.	Comment.
1	BB-TxWin	Broadband PLC system with windowing at transmission side only.
2	BB-RxWin	Broadband PLC system with windowing at reception side only.
3	BB-dbWin	Broadband PLC system with double windowing: at transmission and reception side.
4	BB-dbWin-max	Broadband PLC system double windowing: at transmission and reception side but with maximun RI’ value.

**Table 5:** NB schemes to be analysed

#	Abbrev.	Comment.
1	NB-TxWin	Narrowband PLC system with windowing at transmission side only.
2	NB-RxWin	Narrowband PLC system with windowing at reception side only.
3	NB-dbWin	Narrowband PLC system with double windowing: at transmission and reception side.
4	NB-dbWin-max	Narrowband PLC system double windowing: at transmission and reception side but with maximun admissible RI’ value.

**Table 6:** Main parameters of physical layer of CENELEC-A.

Parameter	Description	Value (Samples)
*N*	Size of FFT	256
*M*	Active carriers †	36
μ	Length of CP	30
RI	Samples of roll-off interval in Tx.	8

† Active subcarriers occupy consecutive positions from $N_{23}$ to $N_{58}$.

## Data Availability

Data sharing not applicable.
